# Supplementary material for: 89Zr-Labeled Domain II-Specific scFv-Fc ImmunoPET Probe for Imaging Epidermal Growth Factor Receptor In Vivo
Source: Cancers (Basel). 2021 Feb 1;13(3):560. doi: 10.3390/cancers13030560 (PMC7867132; doi:10.3390/cancers13030560)
Supplement: Supplementary file 1 [file cancers-13-00560-s001.pdf]

cancers

MDPI

Article

^{89}Zr-labeled Domain II-Specific scFv-Fc ImmunoPET Probe for Imaging Epidermal Growth Factor Receptor In Vivo

Elahe Alizadeh [1], Khan Behlol Ayaz Ahmed [1], Viswas Raja Solomon [1], Vijay Gaja [1], Wendy Bernhard [2], Amal Makhlouf [1,3], Carolina Gonzalez [2], Kris Barreto [2], Angel Casaco [4], C. Ronald Geyer [2,*] and Humphrey Fonge [1,5,*]

[1] Department of Medical Imaging, University of Saskatchewan, College of Medicine, Saskatoon SK S7N 0W8, Canada; elahe.alizadeh@queensu.ca (E.A.); khanbehlol@gmail.com (K.B.A.A.); vrajasolomon@gmail.com (V.R.S.); vijay.gaja@lightsource.ca (V.G.); amal.makhlouf@pharma.cu.edu.eg (A.M.)
[2] Department of Pathology and Laboratory Medicine, University of Saskatchewan, College of Medicine, Saskatoon SK S7N 5E5, Canada; wlb302@mail.usask.ca (W.B.); cag948@mail.usask.ca (C.G.); kmb107@mail.usask.ca (K.B.)
[3] Department of Pharmaceutics and Industrial Pharmacy, Faculty of Pharmacy, Cairo University, Cairo 11562, Egypt
[4] Centre for Molecular Immunology, 11600, Havana, Cuba; casaco@cim.sld.cu
[5] Department of Medical Imaging, Royal University Hospital (RUH), Saskatoon SK S7N 0W8, Canada.
* Correspondence: humphrey.fonge@usask.ca (H.F.); Tel.: 306-655-3353; Fax.: 306 655 1637; ron.geyer@usask.ca (C.R.G.); Tel: 306-966-2040

Supplementary Figures

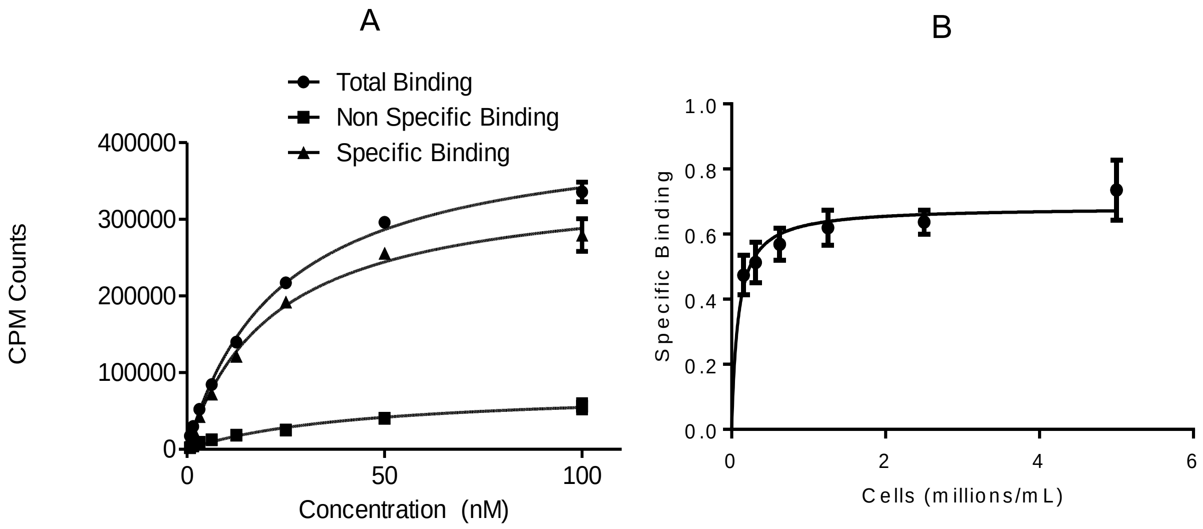

Figure 1. A) Saturation radioligand binding assay of ^{89}Zr-8709-scFv-Fc in EGFR positive DLD-1 cells. B) Immunoreactive assay for ^{89}Zr-8709-scFv-Fc in EGFR positive DLD-1 cells.

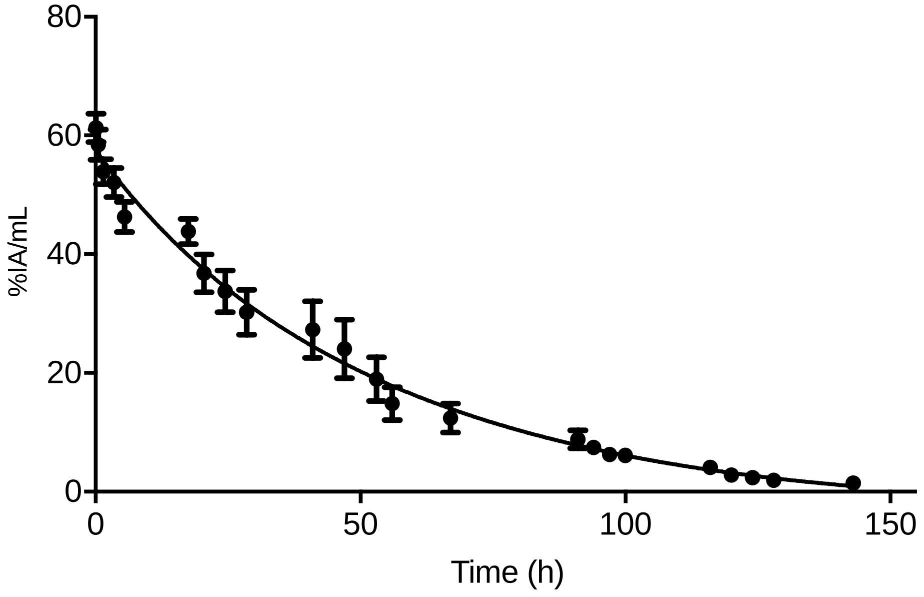

Figure 2. Pharmacokinetics of ^{89}Zr-8709-scFv-Fc in healthy Balb-C mice. Blood values were expressed as % injected activity per mL (% IA/mL) +/- SEM.

a)

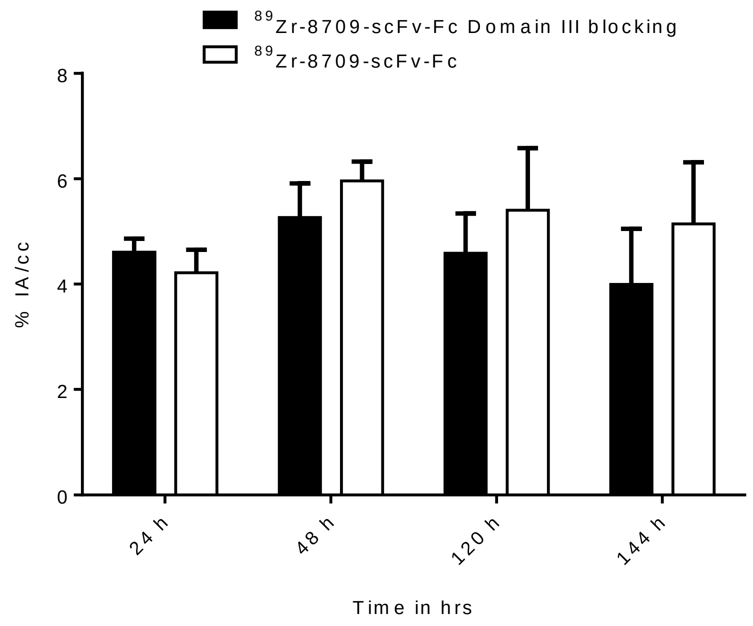

b)

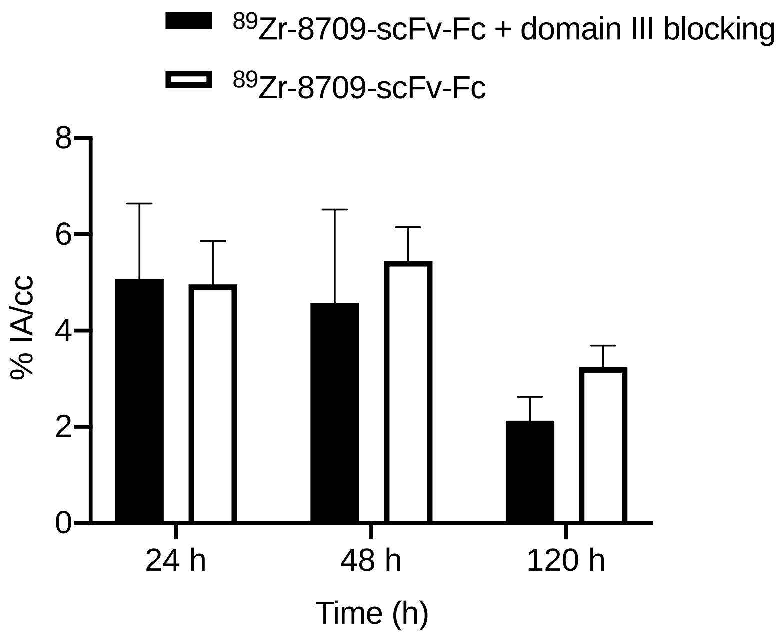

Figure 3. Tumor ROI quantification of microPET/CT images of mice pre-injected with nimotuzumab (domain III) prior to administration of ^{89}Zr-8709-scFv-Fc. a) DLD-1 tumor bearing mice pre-dosed with nimotuzumab followed by ^{89}Zr-8709-scFv-Fc, and b) MDA-MB-231 tumor bearing mice pre-dosed with nimotuzumab followed by ^{89}Zr-8709-scFv-Fc

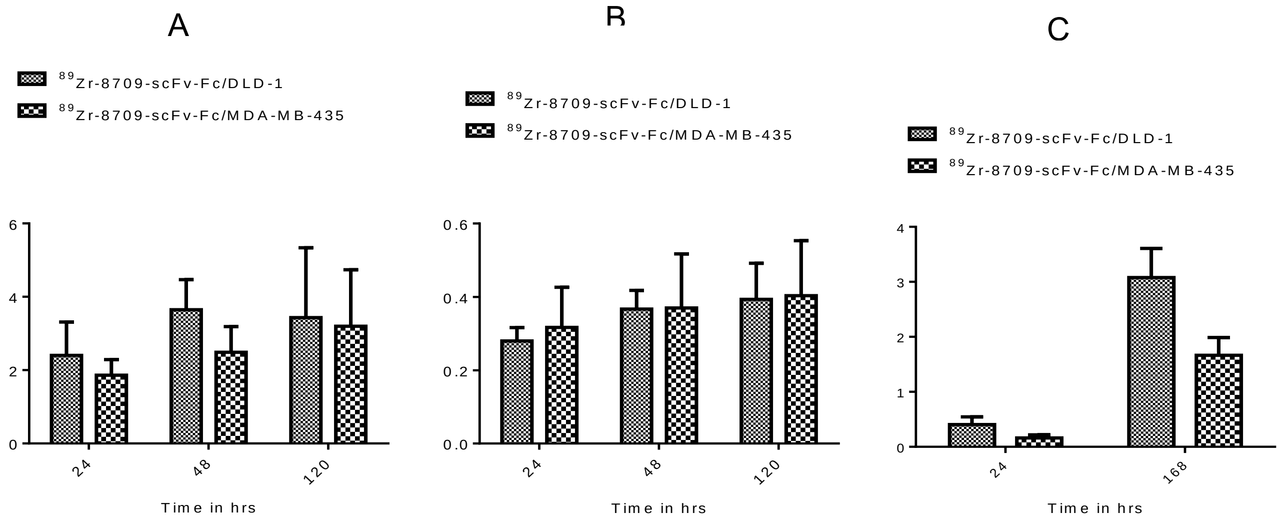

Figure 4. Tumor to normal organ ratios of ^{89}Zr-8709-scFv-Fc at different times post-injection (using microPET/CT image analysis (A & B) and biodistribution data (C)). A) Tumor/muscle ratio. B) Tumor/liver ratios. C) Tumor/blood (data from biodistribution).
